# Supplementary material for: A Critical View of the Application of the APEX Software (Aqueous Photochemistry of Environmentally-Occurring Xenobiotics) to Predict Photoreaction Kinetics in Surface Freshwaters
Source: Molecules. 2019 Dec 18;25(1):9. doi: 10.3390/molecules25010009 (PMC7017383; doi:10.3390/molecules25010009)
Supplement: Supplementary file 1 [file molecules-25-00009-s001.zip › 3.2.4_gcc-4.4.0.zip!/3.2.4_gcc-4.4.0/3.2.4_gcc-4.4.0/doc/octave/html/interpreter/Interacting-with-plots.html]

Interacting with plots - Untitled


Next: Test Plotting Functions,
Previous: Printing Plots,
Up: Plotting Basics


---

#### 15.1.7 Interacting with plots

The user can select points on a plot with the `ginput` function or
selection the position at which to place text on the plot with the
`gtext` function using the mouse.

— Function File: [x, y, buttons] = **ginput** (n)  
> Return which mouse buttons were pressed and keys were hit on the current
> figure. If n is defined, then wait for n mouse clicks
> before returning. If n is not defined, then `ginput` will
> loop until the return key is pressed.

— Function File: b = **waitforbuttonpress** ()  
> Wait for button or mouse press.over a figure window. The value of
> b returns 0 if a mouse button was pressed or 1 is a key was
> pressed.
>
>
>
> **See also:** ginput.

— Function File: **gtext** (s)  
— Function File: **gtext** ({s1; s2; ...})  
— Function File: **gtext** (..., prop, val)  
> Place text on the current figure using the mouse. The text is defined
> by the string s. If s is a cell array, each element of the cell
> array is written to a separate line. Additional arguments are passed to
> the underlying text object as properties.
>
>
>
> **See also:** ginput, text.
